# Supplementary figures and images for: Are reaching and grasping effector-independent? Similarities and differences in reaching and grasping kinematics between the hand and foot
Source: Exp Brain Res. 2022 Apr 15;240(6):1833–48. doi: 10.1007/s00221-022-06359-x (PMC9142431; doi:10.1007/s00221-022-06359-x)

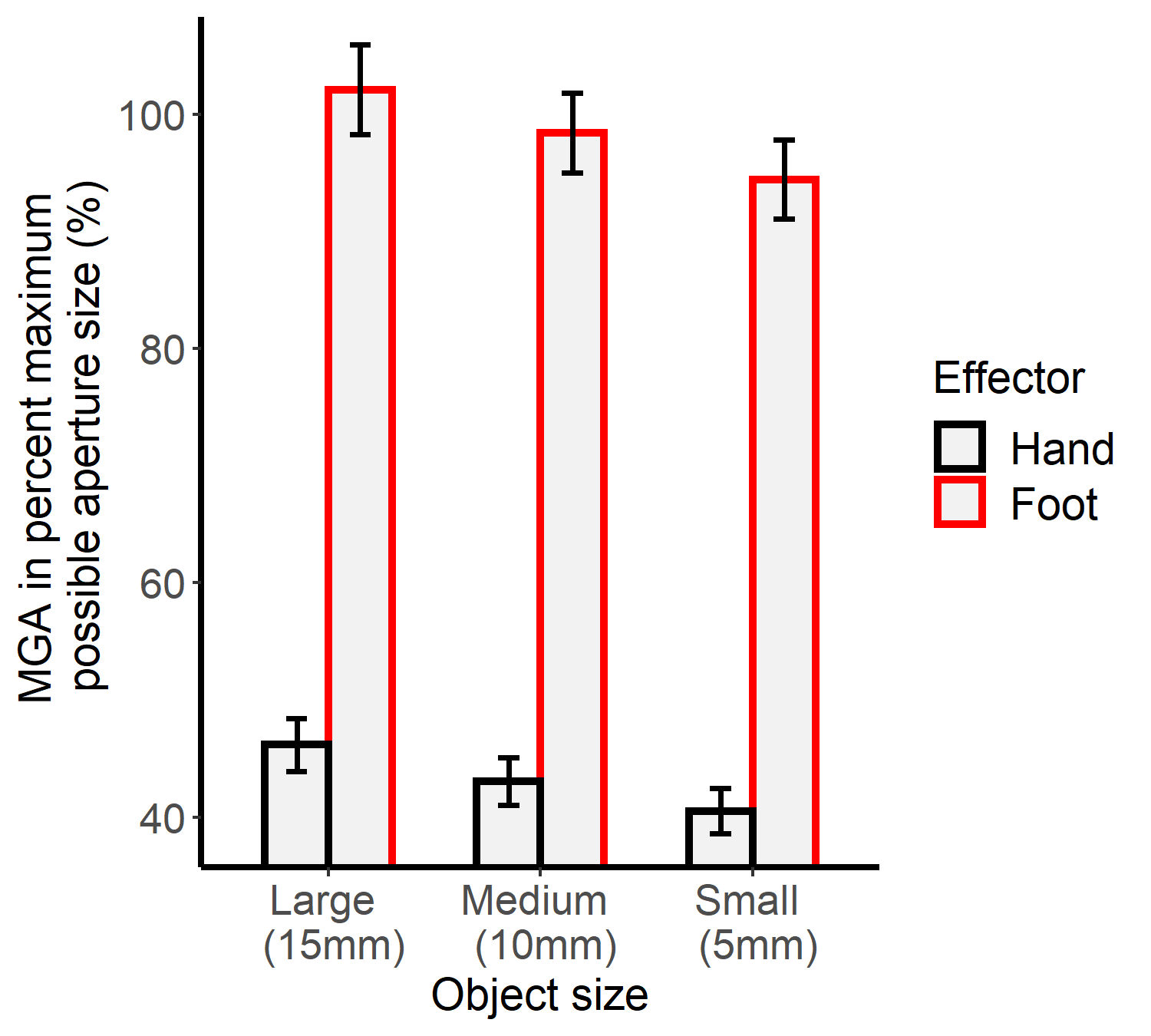

Supplement: Supplementary file 2 — Supplementary file2 (PNG 34 KB) [file 221_2022_6359_MOESM2_ESM.png]
